# Stability characteristics of medial meniscus tear in mild varus knee: a finite element analysis

**DOI:** 10.1186/s12891-025-09046-4

**Published:** 2025-10-09

**Authors:** Chengyue Yu, Wenjun Zhao, Kexin Liu, Xiaoyuan Duan, Xiaokang Gao, Lupeng Wang, Jinwei Liu, Jiahe Xu, Jiashi Zeng, Guangdong Chen, Desheng Chen, Guosheng Xing, Weiguo Xu

**Affiliations:** 1 Tianjin Hospital, Tianjin University, Tianjin, China; 2 Weifang People’s Hospital, Weifang, Shandong China; 3 Department of Orthopedics, North China Medical Health Group Fengfeng General Hospital, Handan, Hebei China; 4 Clinical School, College of Orthopedics, Tianjin Medical University, Tianjin, China; 5 Academy of Medical Engineering and Translational Medicine, Tianjin University, Tianjin, China; 6 Department of Orthopaedics, Cangzhou Central Hospital, Cangzhou, Hebei China

**Keywords:** Knee joint, Mild varus knee, Meniscus tear, Healing potential, Finite element analysis

## Abstract

**Background:**

Recent studies have pointed out that varus alignment is a potential risk factor for medial overload and the development of osteoarthritis, which may influence the healing of meniscus tears. This study aimed to analyze the stress distribution of radial and longitudinal meniscal tears in mild varus knee and use it to explore the effects of varus alignment, tear type and length on the healing potential of medial meniscal tears.

**Methods:**

A healthy volunteer was recruited, and computed tomography (CT) and magnetic resonance imaging (MRI) scans of the right knee were performed to develop a subject-specific three-dimensional finite element model at a neutral position of 0° and with tibial varus angles of 3°, 6°, and 9°. The model contained bone structures (femur, tibia, fibula) and soft tissues (menisci, cartilage, ligaments). Stable and unstable radial tears and stable and unstable longitudinal tears (located in the white, red-white, or red zones) were introduced in the posterior medial meniscus, followed by finite element analysis.

**Results:**

The peak contact pressure in the medial compartment increased linearly with increasing varus angle (Slope = 1.3599, R^2^ = 0.99223). When the varus increases by 1°, the corresponding peak contact pressure increased by an average of 0.46 MPa. In the case of varus knee, the maximum stress in radial tears was localized at the tear apex. Additionally, the stress of unstable radial tears was higher than that of stable radial tears. The stress distribution on the inner and outer surfaces of longitudinal tears depended on the varus alignment and the tear position. With increasing varus angle, stable longitudinal tears in the red-white and white zones showed a gradual decrease in favourable stress differences(White zone: 0.39 MPa→0.20 MPa; Red-white zone: 0.53 MPa→0.36 MPa), while unfavourable stress differences emerged (White zone: 0.20 MPa→-0.40 MPa, while unfavourable stress differences emerged (White zone: 0.20 MPa→-0.40 MPa; Red-white zone: 0.36 MPa→-0.43 MPa). Unstable longitudinal tears in the white zone exhibited increased unfavourable stress differences(-0.18 MPa→-0.52 MPa). Notably, unstable longitudinal tears consistently demonstrated unfavourable stress differences even under mild varus conditions.

**Conclusion:**

To our knowledge, this study is among the first to investigate the influence of varus alignment on stress distribution in meniscal tears using finite element analysis. This study investigates two types of radial tears and two types of longitudinal tear prototypes in three zones simultaneously. This study provides novel insights into how varus alignment influences stress distribution in meniscal tears, suggesting its potential role in guiding treatment decisions. Future studies incorporating clinical data are warranted to validate these findings.

**Supplementary Information:**

The online version contains supplementary material available at 10.1186/s12891-025-09046-4.

## Introduction

The meniscus plays a pivotal role in maintaining normal knee joint function. It serves to protect articular cartilage by load transmission, shock absorption, and increasing joint contact area [1]. Meniscal tears, among the most prevalent knee injuries, are commonly associated with articular cartilage degeneration and frequently progress to early-stage osteoarthritis [2, 3].

Meniscal tears exhibit diverse morphological patterns with distinct etiologies. Radial tears are usually perpendicular to the edge and extend to the periphery, resulting in joint wear and aggravating degenerative lesions [4]. Meniscectomy is often used for treatment. Longitudinal vertical tears typically originate at the posterior horn, progressively extending toward the meniscal body, and are frequently observed in physically active young individuals [3, 5]. Treatment methods usually use meniscectomy or repair, and the most suitable repair is the longitudinal vertical tear that occurs in the red zone [5, 6]. Meniscal repair effectively restores meniscal function and has been shown to yield positive long-term clinical results. On the one hand, this is due to the vascular factor; on the other hand, the biomechanics of the meniscus also play a role [7].

Current treatment strategies for meniscal tears have shifted from meniscectomy toward surgical repair and non-surgical treatment, primarily due to reliance on the meniscus’s intrinsic healing capacity [6]. Previous studies have demonstrated that both the type and location of meniscal tears influence their healing potential [7]. However, current studies have been limited to neutral position and flexion, and it is not yet clear whether the varus alignment influences the healing potential of the meniscal tear. Clinically, varus alignment is a recognized risk factor for medial compartment overload and osteoarthritis progression [8, 9], with evidence suggesting its negative effect on degenerative tear healing [10]. Biomechanical studies consistently show increased peak contact pressure in the medial compartment under varus conditions [8, 9, 11]. Additionally, research has shown that longitudinal tears in the lateral meniscus exhibit significant expansion under valgus torque [12]. However, while biomechanical experiments provide valuable insights into knee mechanics, they are limited in their ability to elucidate the mechanisms underlying meniscal tear healing. In contrast, finite element simulations provide intuitive explanations for visualizing and interpreting these complex biomechanical processes. Therefore, the purpose of this study was to establish a mild tibial varus knee model to analyze the stress distribution of longitudinal tear and radial tear under mild varus conditions. We hypothesized that the varus alignment would influence the healing potential of meniscal tears.

## Materials and methods

This study recruited a 22-year-old male volunteer (Height : 170 cm, Body Mass Index (BMI) : 23, Hip-Knee-Ankle angle (R_HKA) : 0.44°, Mechanical medial proximal tibial angle (R_mMPTA) : 88.61°, Mechanical lateral distal tibial angle (R_mLDTA) : 87.07°, Mechanical lateral distal femoral angle (R_mLDFA) : 88.77°, Joint line convergence angle (R_JLCA) : 0.30°). The volunteer had no history of medical or surgical diseases and no history of knee joint injury or operation. Physical and X-ray examinations ruled out knee joint diseases. The volunteer was provided with an informed consent for the study and signed the informed consent form. All methods in this study were carried out in accordance with the Helsinki Declaration. The experimental protocols of this study were approved by the Ethics Committee of Tianjin University Hospital, Tianjin, China (IRB: 2024 Medical Ethics Bureau Pre-020).

### Data acquisition

The volunteer’s right knee joint was selected for magnetic resonance imaging (MRI) (SIGNA 3.0T; GE, Las Vegas, NV, USA) with a slice thickness of 1 mm, and a total of 250 MRI slices were obtained to obtain soft tissue (meniscus, ligaments, and articular cartilage) from sagittal T1WI MRI, sagittal T2WI MRI and axial T2WI. The same part of the volunteer was scanned with computed tomography (CT) using the GE Discovery CT 750 HD device with a slice distance of 0.625 mm to obtain bone tissue (femur, tibia, fibula, and patella).

### Three-dimensional modeling of the knee joint

The DICOM images of the knee joint acquired by CT and MRI were imported into MIMICS (Materialise, Leuven, Belgium) software to reconstruct the bone and soft tissue structures. Segmentation was performed based on grayscale values. The femur, tibia, fibula, and patella were segmented from the CT images. The cartilage (femoral cartilage, tibial cartilage, fibular cartilage, and patellar cartilage), meniscus (medial meniscus, lateral meniscus), ligaments (anterior cruciate ligament (ACL), posterior cruciate ligament (PCL), medial collateral ligament (MCL), lateral collateral ligament (LCL)), and quadriceps tendon were segmented from the MRI images. To ensure the accuracy of the connection position of each tissue, the subjects kept the same posture as much as possible during data acquisition. The knee joint’s bone structure and soft tissue were manually segmented by experienced orthopedic clinicians and radiologists.

**Fig. 1:**
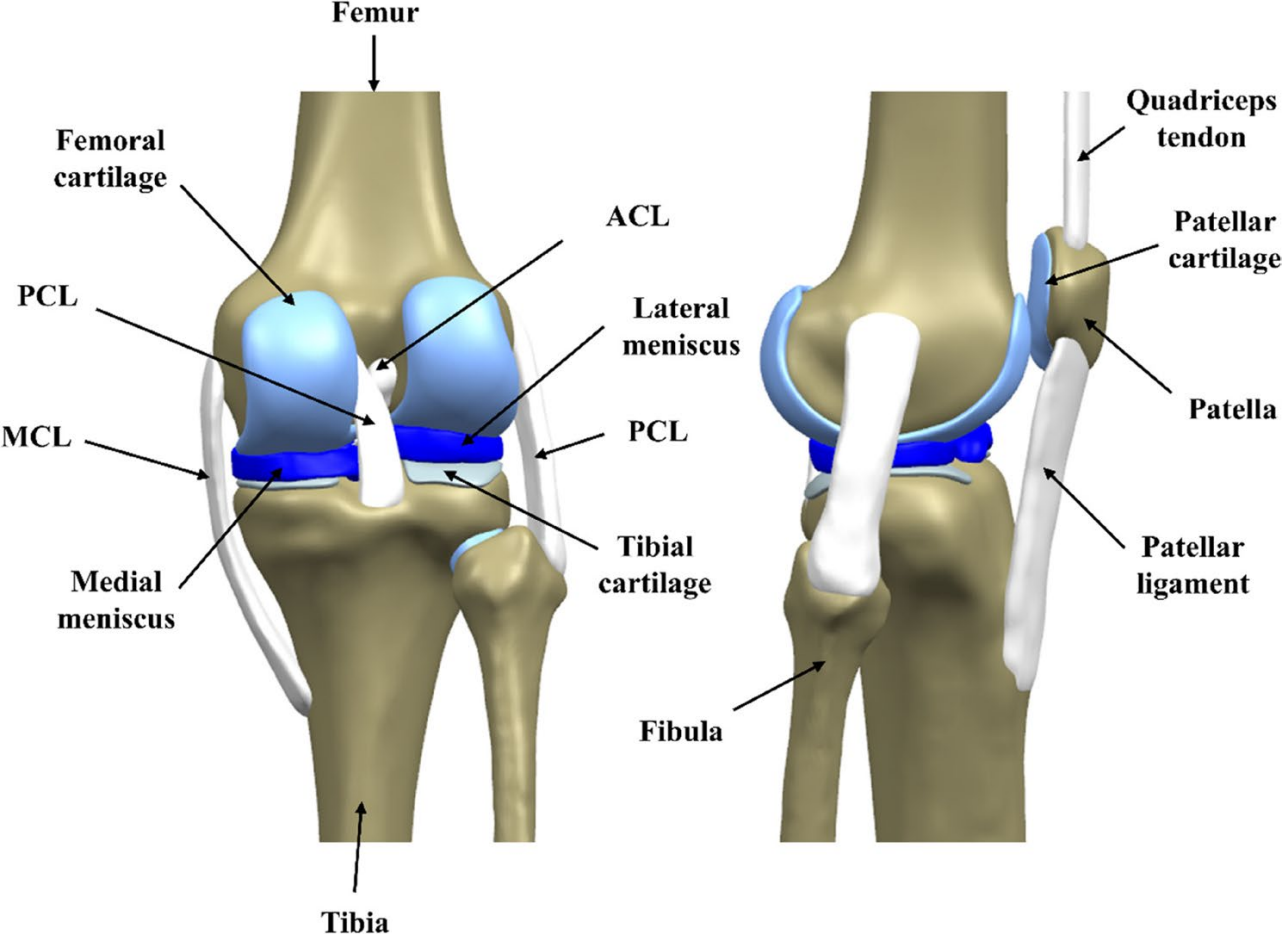
Schematic diagram of knee joint structure

Each knee joint model was smoothed, corrected, and surface-fitted using Geomagic Studio (Geomagic Solutions, Cary, NC, USA). The soft tissue generated by the MRI image and the bone structure generated by the CT image was imported into Solidworks software and combined. All structures were precisely integrated according to their specific anatomical positions, and the surface resection function in the software was used to make the meniscus articular surface fit closely and accurately in contact with the femoral and tibial articular surfaces. This step was performed under the guidance of an experienced orthopedic clinician. The three-dimensional (3D) model of the knee joint constructed in this step is shown in Fig. 1:

### Finite element model of knee joint

**Fig. 2:**
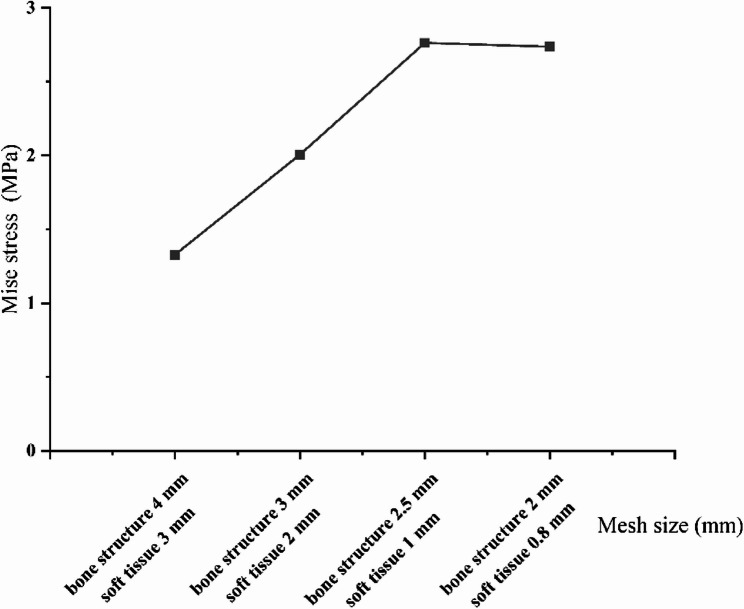
Mesh convergence plot

The 3D geometric model of the knee joint was imported into Abaqus 6.14 (SIMULIA, France) and calculated using the Standard solver. The knee joint’s complete finite element model was established through meshing, material assignment, and boundary condition setting. The model uses linear tetrahedral meshes. After setting the mesh size, mesh independence verification is also required. The error of the peak stress of the tibial cartilage was within 2%, which is acceptable for establishing multiple grid models from coarse to fine, as shown in Fig. 2. The bone structure unit size is 2.5 mm, and the average soft tissue unit size is 1 mm. The neutral knee joint model has 773,394 tetrahedral units and 201,499 nodes.

### Material properties

Material properties can accurately describe the mechanical behavior of tissues and are crucial to the accuracy of the results. Cortical bone material was an isotropic elastic material with an elastic modulus (*E*) of 17,000 MPa and a Poisson’s ratio ($\nu$) of 0.3 [13]. Cancellous bone material was also an isotropic elastic material with an elastic modulus (*E*) of 400 MPa and a Poisson’s ratio($\nu$) of 0.3 [14].The Yeoh model accurately expressed the material properties of articular cartilage and simulated the isotropic, hyperelastic, almost incompressible behavior of articular cartilage [15, 16], as detailed in Supplementary Material. Meniscus material properties were defined based on constructed as transversely isotropic hyperelastic material [17–19], by combining the strain energy density function with the Holzapfel-Gasser-Ogden (HGO) material model, as detailed in Supplementary Material. Ligaments material properties were defined based on established nearly incompressible, nonlinear, hyperelastic, transversely isotropic fiber material [20, 21], as detailed in Supplementary Material.

### Establishment of mild varus knee model and meniscus tear model

In this study, we used tibial varus (HKA ≤ 10°, mMPTA < 87°±3°). Firstly, a neutral 0° knee joint model should be established, and on this basis, 3°, 6°, and 9° varus knee models should be established based on the HKA and mMPTA angle through the tibia source. This study investigates the potential impact of varus alignment on stress distribution patterns across meniscal tear surfaces.

**Fig. 3:**
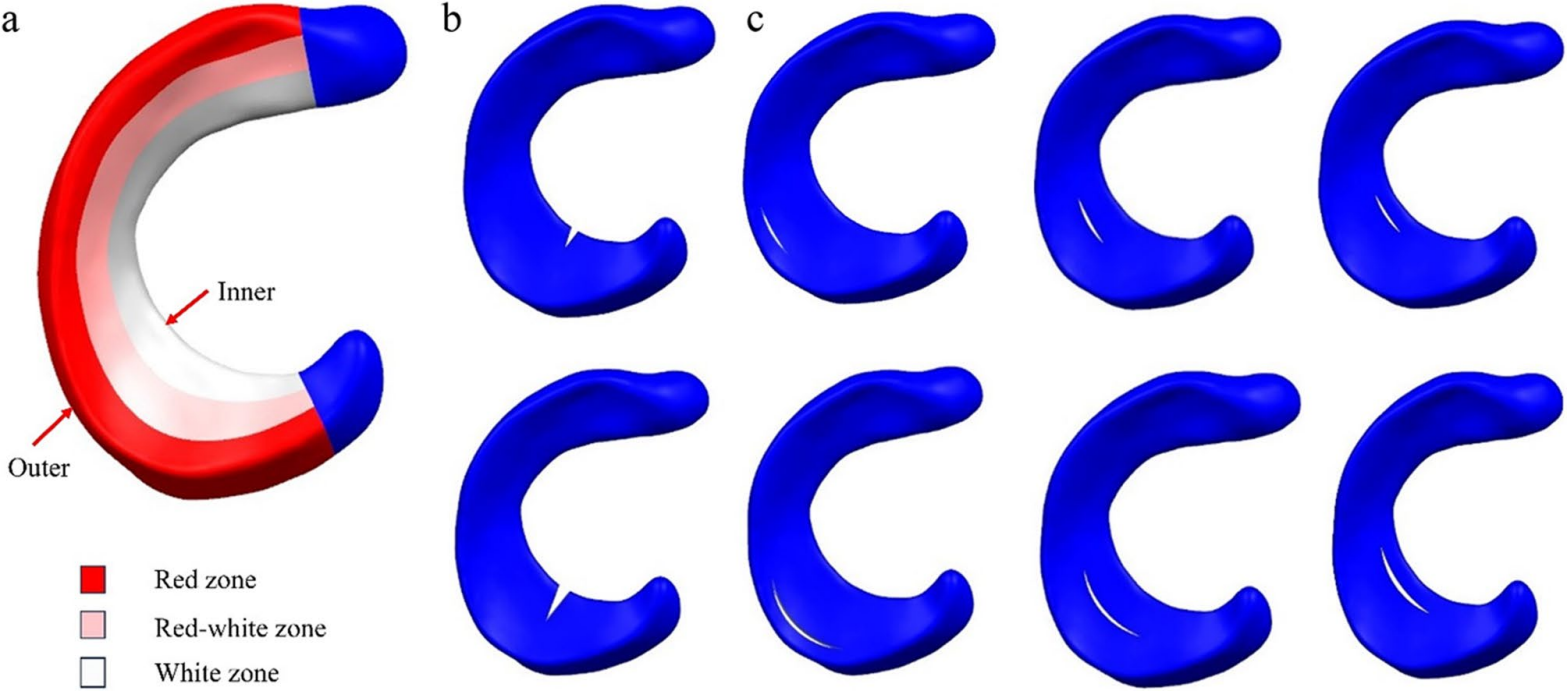
**a** Red zone, red-white zone, and white zone of the meniscus. **b** Stable and unstable radial tears in the posterior region of the meniscus. **c** Stable and unstable longitudinal tears in the posterior region of the meniscus occurring in the red zone, red-white zone, and white zone

Based on the aforementioned model, meniscal tears model were subsequently established. Studies have found that the injury rate of the medial meniscus is much higher than that of the lateral meniscus [22], with the posterior half bearing greater functional loads. Therefore, the tear area in this study was set in the posterior half of the medial meniscus. Kedgley et al. [7] mentioned that stable radial tears were defined as those shorter than one-third of the width of the meniscus, and stable vertical longitudinal tears were defined as tears less than 10 mm. In this study, we built tear models using the complete meniscus model in SolidWorks software and then imported it into Abaqus software for meshing. The following settings established its type, length, and position: stable radial tears were located in the white zone of ​​the meniscus, and the length occupies 1/4 of the width of the meniscus. Unstable radial tears start from the white zone of ​​the meniscus and extend to the red-white zone, and the length occupies 1/2 of the width of the meniscus. The length of the stable longitudinal tear is 9 mm, and the length of the unstable longitudinal tear is 15 mm. Its location was set in the red zone, red-white zone, and white zone of ​​the meniscus. This is shown in Fig. 3. Defines normalized length, the process of normalizing the length from the inner edge to the outer edge of the meniscus, scaling the length value to between 0 and 1. The Mise stress sampled from the inner edge (Normalized length = 0) to the outer edge (Normalized length = 1) of the meniscus was used as an indicator of hoop stress. When the stress on the inner surface of the tear was greater than the stress on the outer surface, a favourable stress difference existed (${S_{{\text{inner}}}}> {S_{outer}}$). When the stress on the inner surface of the tear was less than the stress on the outer surface, an unfavourable stress difference existed (${S_{{\text{inner}}}}< {S_{outer}}$).

### Loads and boundary conditions settings

In this study, the intact knee joint containing the ligaments was established. Boundary conditions were defined as follows: the lower ends of the tibia and fibula were completely fixed, the femur was free in the axial direction, and the varus-valgus and internal-external rotation angles were free. The contact between the various components of the knee joint was friction contact, and the ligament and bone were rigidly connected. The patella was rigidly connected to the patellar ligament and quadriceps tendon, and a constant load of 40 N was applied above the quadriceps tendon [23]. A tangential slip with a friction coefficient of 0.02 was set between cartilage and cartilage and between cartilage and meniscus [15]. A rigid connection was used between cartilage and bone tissue and between ligament and bone tissue. This study used static mechanical simulation. The value of the force applied was based on the research of Zhang et al. [13]and Zhang et al. [4], applying an axial load of 1150 N to the femur’s upper end to simulate the knee joint’s force in the standing position.

## Results

### Knee joint stress analysis 

#### Meniscus stress analysis

Finite element analysis of the intact meniscus model in neutral position demonstrated higher stress concentrations in the posterior half compared to the anterior half of the medial meniscus. The compressive stress of the posterior horn was 11.38 MPa, and the compressive stress of the anterior horn was 9.429 MPa. The compressive stress of the posterior horn of the lateral meniscus was 5.854 MPa, and the compressive stress of the anterior horn was 4.736 MPa.

**Fig. 4:**
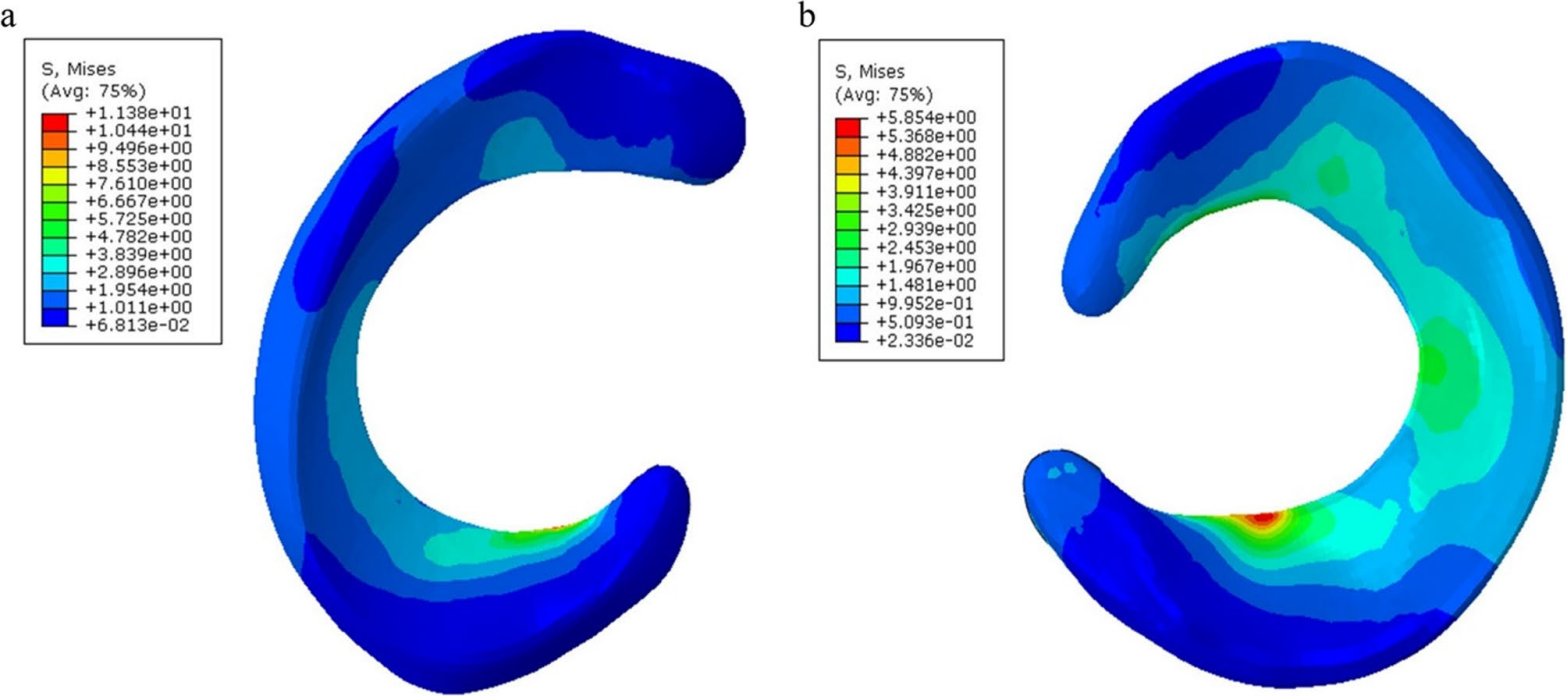
Meniscus compression stress cloud map **a**Medial meniscus **b** Lateral meniscus

The stress of the anterior and posterior horns of the meniscus was higher than that of the body, and the compressive stress of the medial meniscus was greater than that of the lateral meniscus. The maximum stress of the meniscus was located in the posterior root area of ​​the medial meniscus (Fig. 4). The peak contact pressure of the medial compartment of the knee joint was 5.278 MPa, and the peak contact pressure of the lateral compartment was 4.26 MPa.

**Fig. 5:**
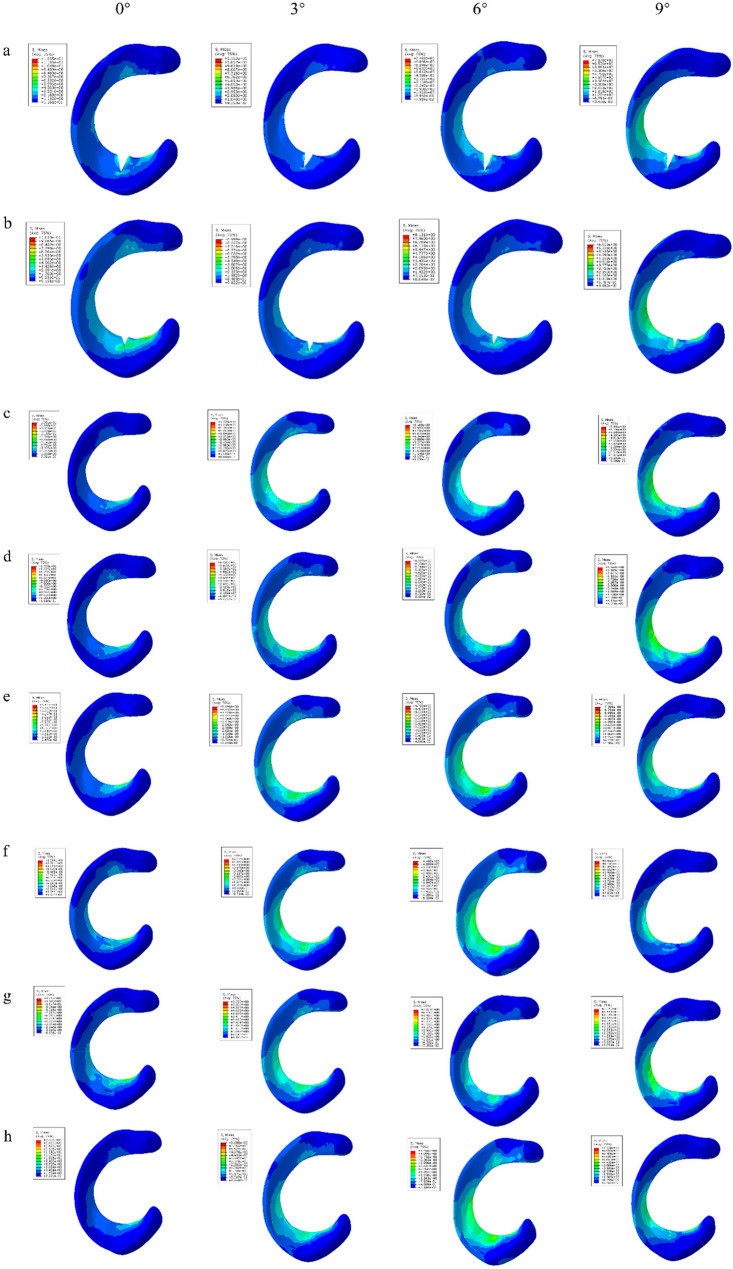
Compressive stress cloud map of meniscus tear in varus knee 0°, 3°, 6° and 9°**a：**Unstable radial tears **b：**Stable radial tears **c：**Stable longitudinal tears in white zone**d：**Stable longitudinal tears in red-white zone **e：**Stable longitudinal tears in red zone **f：**Unstable longitudinal tears in white zone**g：**Unstable longitudinal tears in red-white zone **h：**Unstable longitudinal tears in red zone

Figure 5 displays the compressive stress cloud maps for both stable and unstable radial/longitudinal meniscal tears under 3°, 6°, and 9° varus knee angles. For radial tears, peak stresses consistently localized at the tear apex, with unstable tears exhibiting higher stress than stable counterparts. Longitudinal tears similarly demonstrated peak stress concentrations along both tear margins.

#### Medial compartment peak contact pressure analysis

**Fig. 6:**
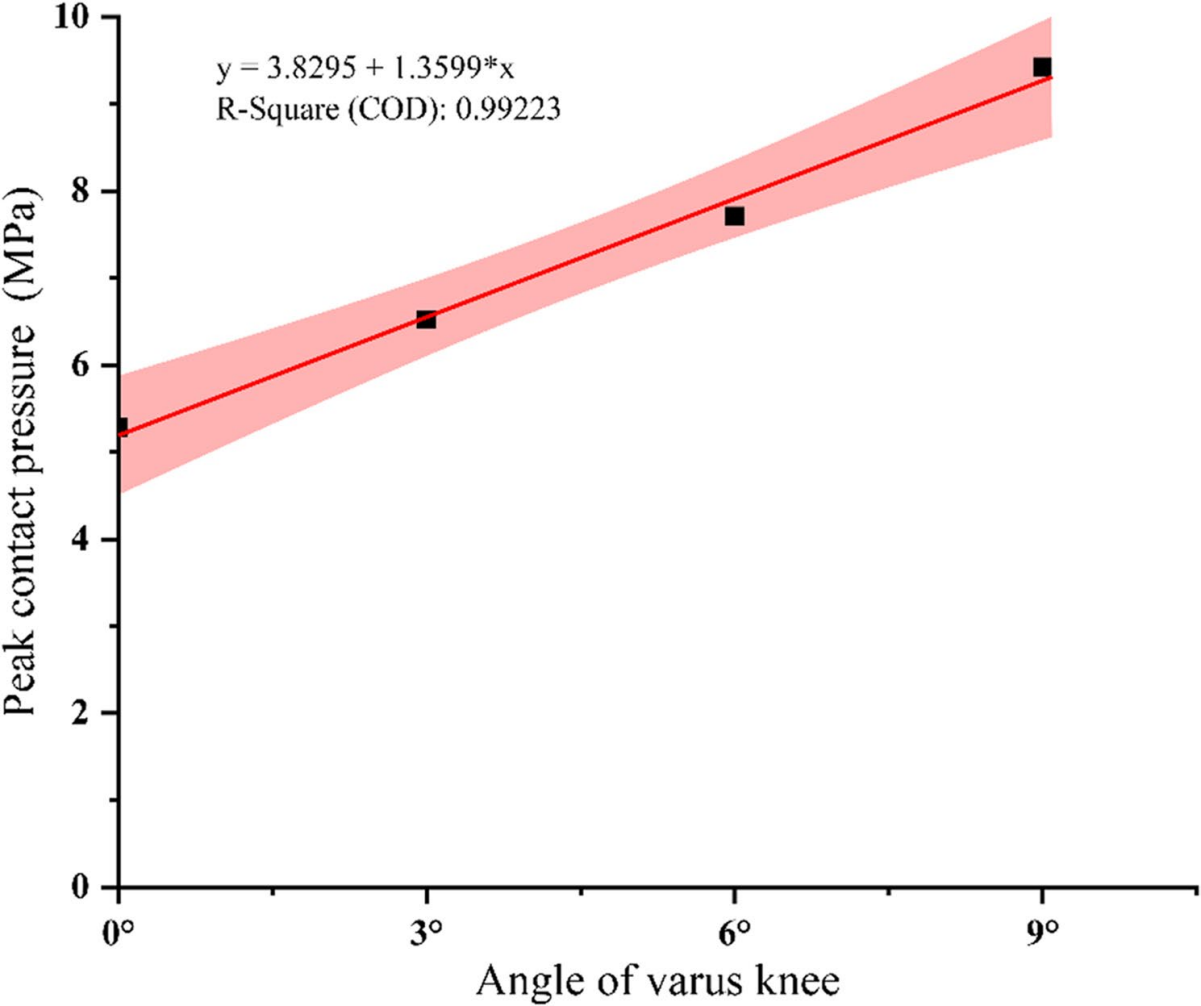
The peak contact pressure of the medial compartment was plotted relative to the angle of the varus knee

The peak contact pressure in the medial compartment from 0° neutral to 9° varus was shown in Fig. 6. The peak contact pressure in the medial compartment increased linearly with increasing varus angle (Slope = 1.3599, R^2^ = 0.99223). When the varus increases by 1°, the corresponding peak contact pressure increased by an average of 0.46 MPa. Compared with the neutral position, the medial compartment pressure increased by 24% (1.24 MPa) at 3° varus, 46% (2.42 MPa) at 6° varus, and 78% (4.14 MPa) at 9° varus.

### Analysis of radial tears

**Fig. 7:**
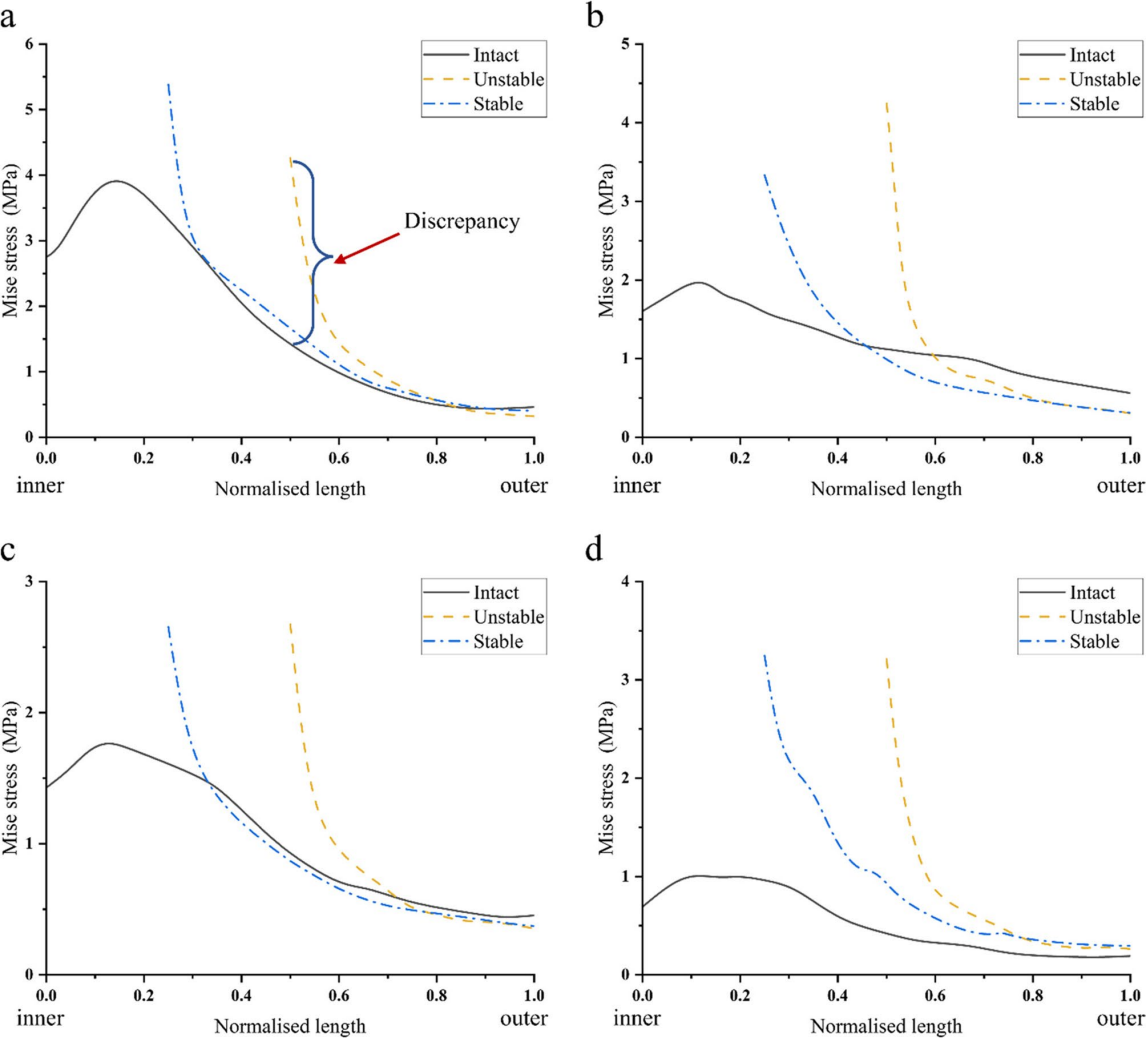
Hoop stress changes from inner to outer edge of intact medial meniscus, stable radial tear, and unstable radial tear at varus knee angle (Normalized length). Differences between the high stresses at the tear apex and the intact condition are indicated by the blue brackets. Normalized length is the process of normalizing the length from the inner edge to the outer edge of the meniscus, scaling the length value to between 0-1. **a**,**b**,**c**, and **d** represent the hoop stress change of the meniscus from the inner edge (Normalized length = 0) to the outer edge (Normalized length = 1) in the neutral position of 0°, varus knee 3°, 6°, and 9°, respectively. A larger stress difference indicates a higher risk of radial tear propagation.

The presence of radial tears destroyed hoop stress distribution in the meniscus, leading to stress concentration at the tear apex. In the 0°, 3°, 6°, and 9° varus knee radial tear of meniscus models, the peak compressive stress in the tear area all appears at the tear apex. Notably, hoop stresses at the tear apex were substantially elevated compared to intact meniscus controls, as shown in Fig. 7.

At different varus knee angles (0°, 3°, 6°, and 9°), the hoop stress of the intact meniscus increased and decreased from the inner to the outer edge. The maximum stress appeared at 10%−20% from the inner edge. This is shown in Fig. 7. There was a gradually decreasing trend from the inner to the outer edge for stable and unstable radial tears. The maximum stress was observed at the inner edge of the meniscus and the minimum stress was observed at the outer edge.

**Fig. 8:**
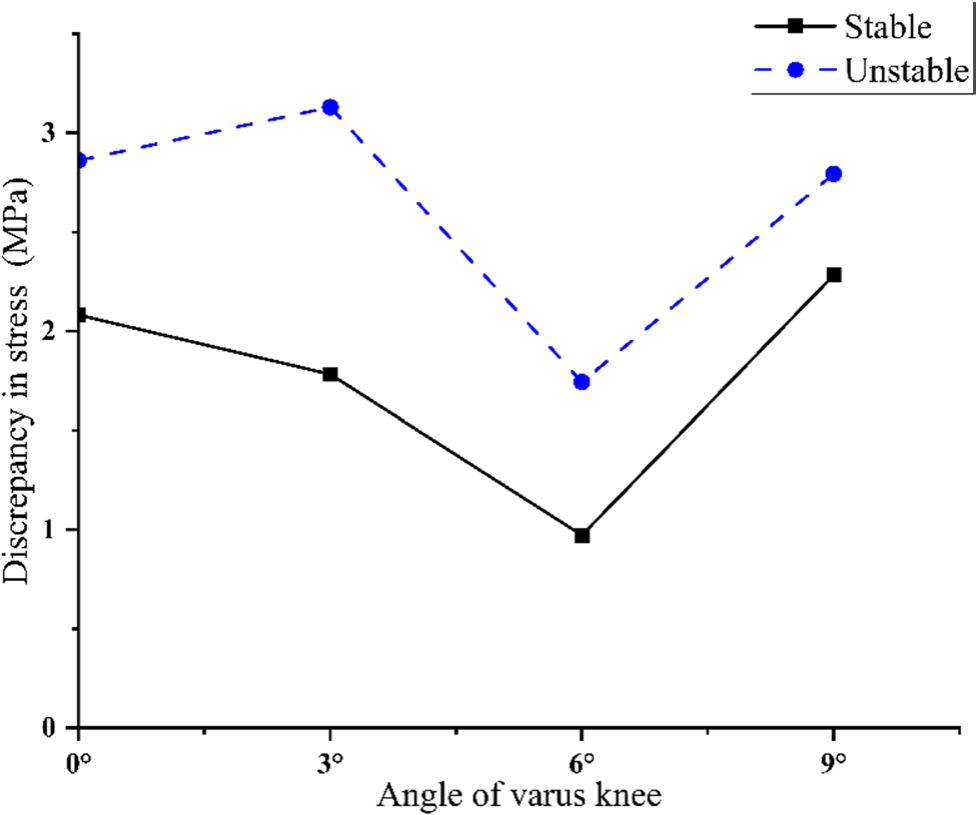
Differences in stress between the apex of stable radial tear and unstable radial tear and the intact meniscus. The stress concentration at the apex of unstable radial tears was significantly higher than that of stable tears at all varus angles, indicating a higher risk of tear propagation

As shown in Fig. 8, there was a stress difference between the crack apex of the radial tear and the intact meniscus, which will cause the tear to move to both sides. The stress difference and the stress gradient change of unstable tears were higher than that of stable tears.

### Analysis of longitudinal tears

**Fig. 9:**
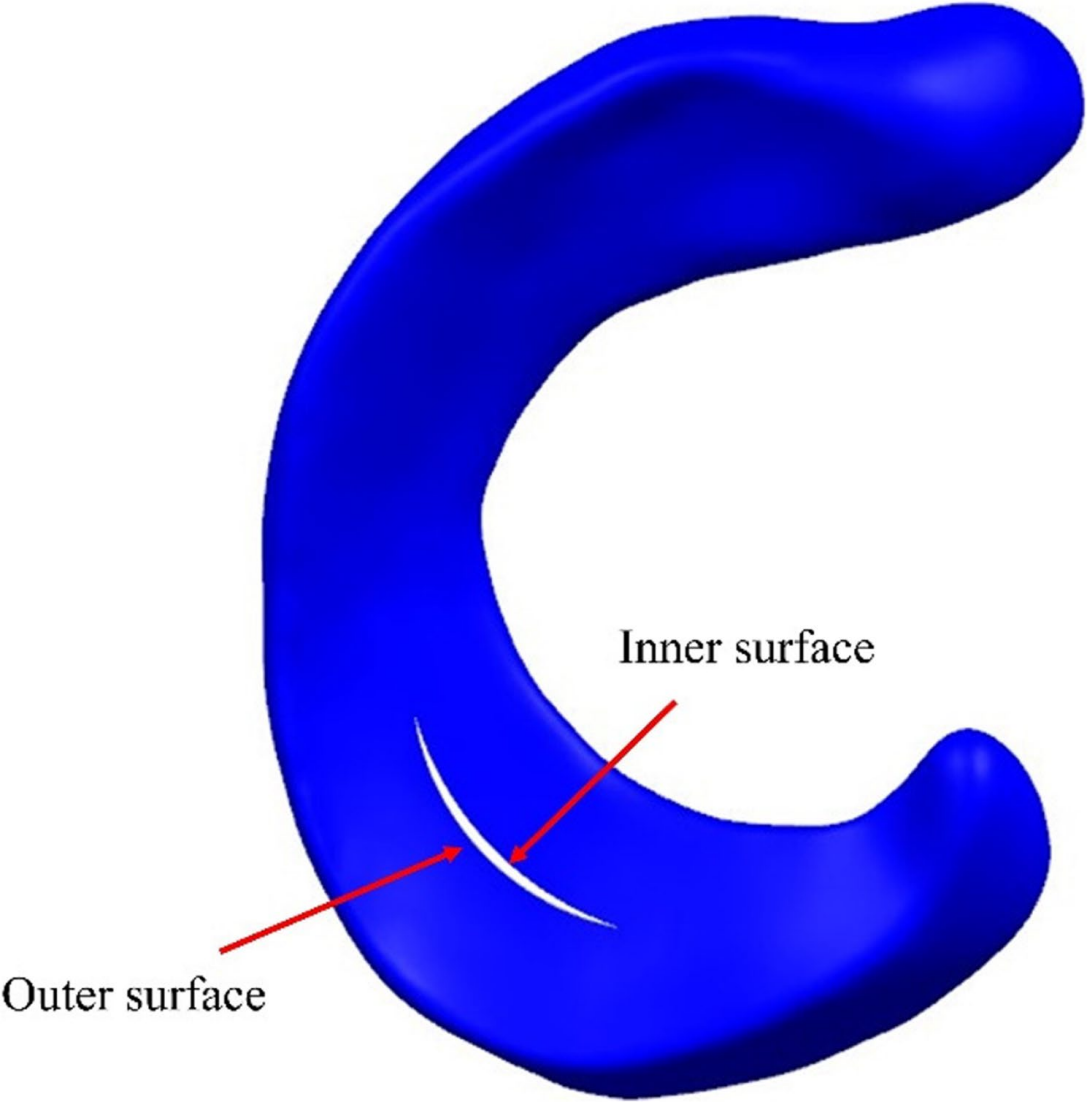
Schematic diagram of the inner and outer surfaces of the medial meniscus

**Fig. 10:**
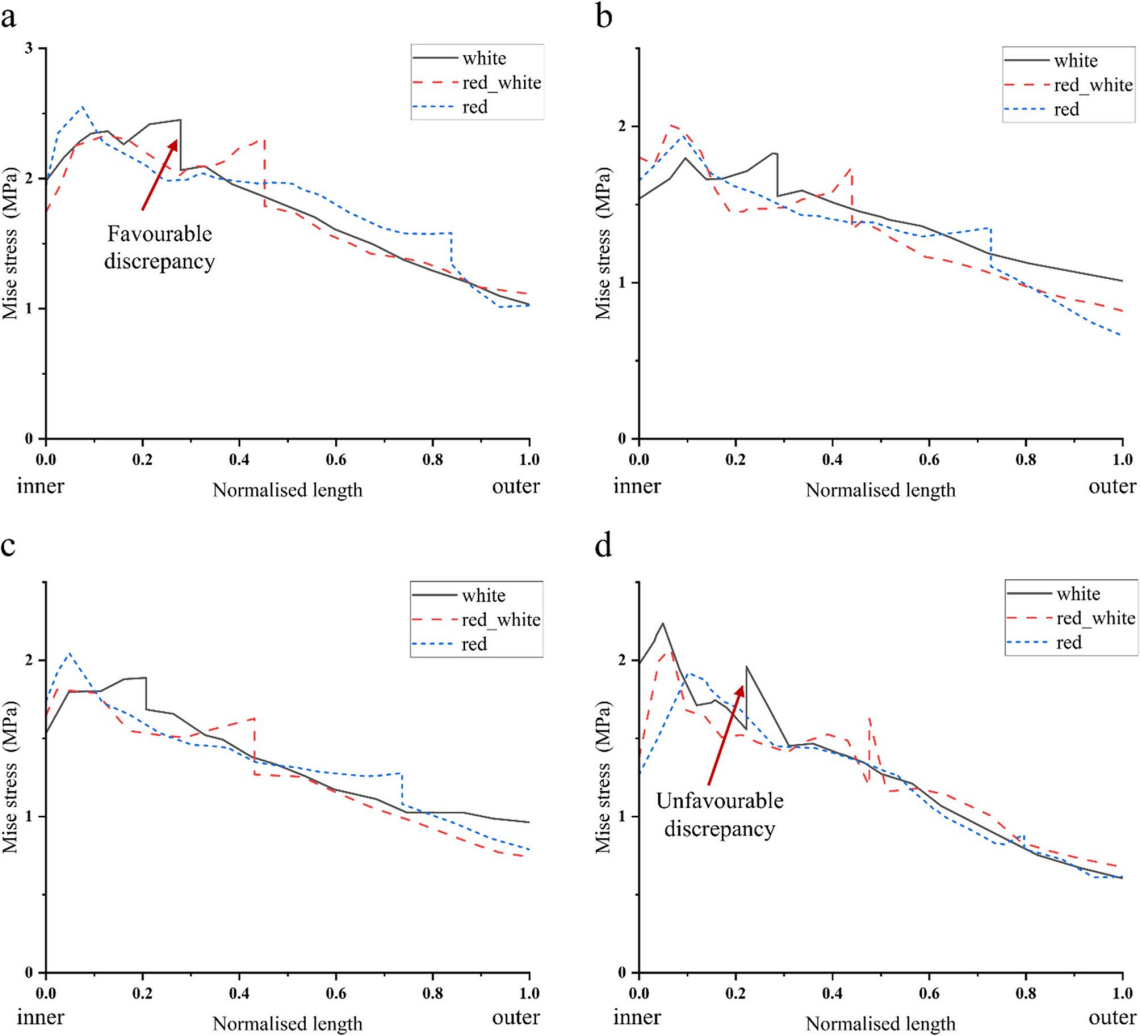
Stress changes of stable longitudinal tears (white zone, red-white zone, red zone) of the meniscus from inner (Normalized length =0) to outer (Normalized length=1).**a**,**b**,**c**,**d **represent the stress changes of the meniscus from the inner edge (Normalized length = 0) to the outer edge (Normalized length = 1) in the neutral position 0°, varus knee 3°, 6°, 9°, respectively. The sharp change in stress indicates the magnitude of the stress difference on the tear surface. A sudden drop in stress indicates that the stress on the inner surface of the tear is greater than the stress on the outer surface, resulting in a favourable stress difference. A sudden rise in stress indicates that the stress on the inner surface of the tear is less than the stress on the outer surface, and there is an unfavourable stress difference.

**Fig. 11:**
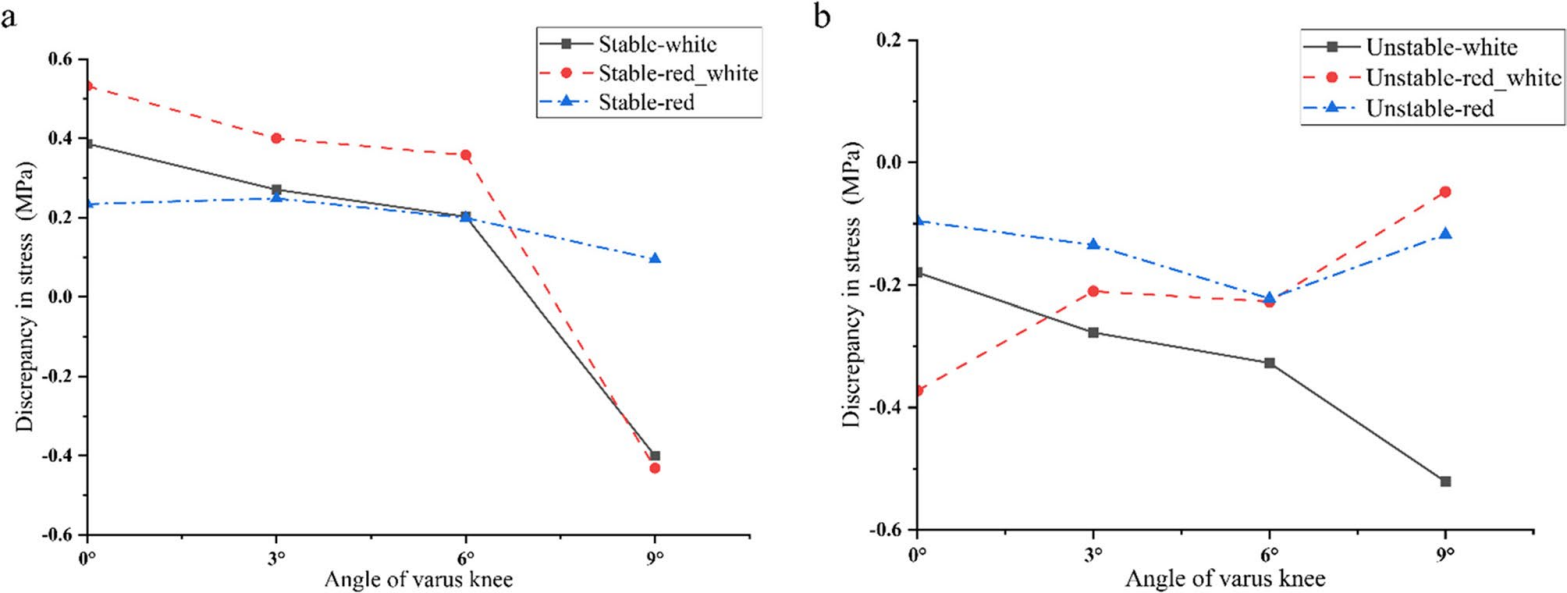
Longitudinal tear surface hoop stress difference plotted relative to varus knee angle. Positive values indicate favourable discrepancy and negative values indicate unfavourable discrepancy **a**: As the varus angle increases, the stable tear stress difference in the red zone is always in a favourable stress state, indicating that the varus angle may have the potential to promote tear healing. The tear stress difference in the white zone and the red and white zones gradually decreases. In the varus 6°−9° stage, an unfavourable stress difference appears, indicating that the varus angle may have the potential to promote tear healing at angles of 0-6° but hinders tear healing at angles of 6-9°. **b**: As the varus angle increases, the unfavourable stress difference of the unstable tear in the white zone increases, indicating that the varus angle may have the potential to hinder tear healing. The unfavourable stress difference of the unstable tear in the red and white zones tends to decrease gradually; however, it has always been in an unfavourable stress state, indicating that the varus angle may have the potential to hinder tear healing.

Under static loading conditions, stable longitudinal tears located in the white zone, red-white zone, and red zone all exhibited outward displacement of the meniscus. At 0°, 3°, and 6° of varus knee, the stress on the inner surface of the tear was greater than the stress on the outer surface at all three positions (Fig. 9), i.e., there was a favourable stress difference, as shown in Fig. 10(a-c). At 9° of varus knee, the stress on the outer surface of the tear was greater than the stress on the inner surface in the white zone and the red-white zone, i.e., there was an unfavourable stress difference, as shown in Fig. 10(d). The stress difference in longitudinal tears located in the red zone remained nearly constant with increasing varus angles. In contrast, tears in the white and red-white zones demonstrated progressively diminishing stress differentials as the varus angle increased (White zone: 0.39 MPa→0.20 MPa; Red-white zone: 0.53 MPa→0.36 MPa). In the varus knee 6°−9° stage, the stress difference decreased sharply, and an unfavorable stress difference appeared (White zone: 0.20 MPa→−0.40 MPa; Red-white zone: 0.36 MPa→−0.43 MPa). This is shown in Fig. 11(a).

**Fig. 12:**
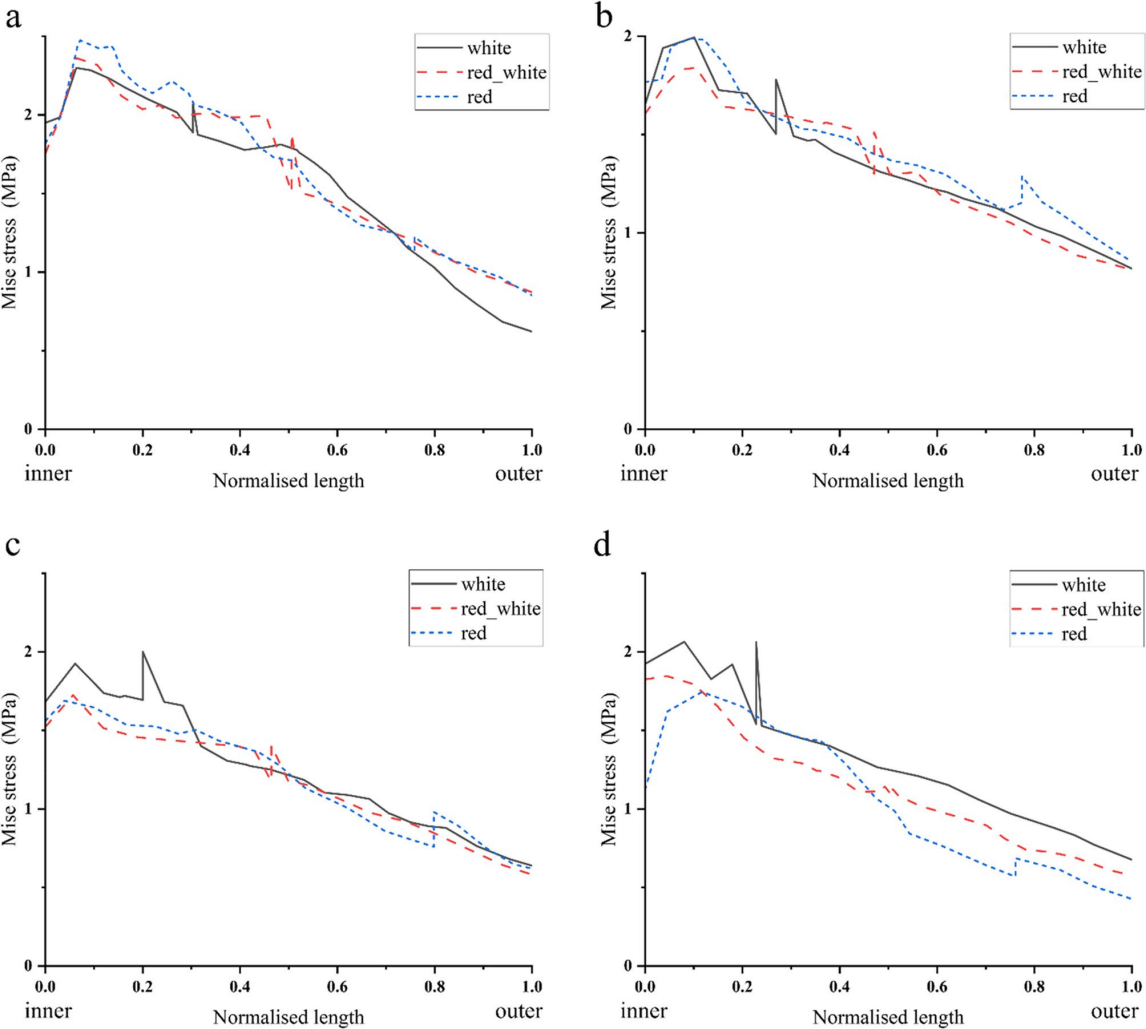
Stress changes of unstable longitudinal tears (white zone, red-white zone, red zone) of the meniscus from inner (Normalized length =0) to outer (Normalized length=1). **a**,**b**,**c**,**d **represent the stress changes of the meniscus from the inner edge (Normalized length = 0) to the outer edge (Normalized length = 1) in the neutral position 0°, varus knee 3°, 6°, 9°, respectively. The sharp change in stress indicates the magnitude of the stress difference on the tear surface. A sudden drop in stress indicates that the stress on the inner surface of the tear is greater than the stress on the outer surface, resulting in a favourable stress difference. A sudden rise in stress indicates that the stress on the inner surface of the tear is less than the stress on the outer surface, and there is an unfavourable stress difference. Unstable longitudinal tears always have unfavourable stress differences on the tear surface when the neutral position is 0°, and varus knee 3°, 6°, and 9, indicating a higher risk of tear propagation.

Unstable longitudinal tears in the white, red-white, and red zones consistently exhibited unfavourable stress differences under mild varus, as shown in Fig. 12 (a-d). With increasing varus angles, the white zone tears showed progressive worsening of unfavourable stress differences (−0.18 MPa→−0.52 MPa). In contrast, the red-white zone tears demonstrated gradually diminishing adverse stress of unfavourable stress differences. Meanwhile, tears located in the red zone demonstrated consistently unfavourable stress differentials with minimal variability. As shown in Fig. 11 (b).

## Discussion

The most important finding of this study was that the varus alignment, the tear type, and the length of the meniscal tear could influence the stress on the meniscal tear surface. While previous studies have demonstrated increased medial compartment pressure with varus alignment, our study extends these findings by highlighting its specific impact on meniscal tear stress distribution.

In this study, we constructed a mild tibial varus knee finite element model including bone, cartilage, ligament, and meniscus to explore the relationship between the tibial varus alignment and the healing potential of meniscal tears. The model validity was verified in the neutral position and mild varus knee. The neutral position intact meniscus model results showed that the peak pressure of the medial/lateral compartment of the knee joint was consistent with the pressure range measured by biomechanical experiments of the specimens [13, 18, 24–26]. The results of the varus knee model showed that the peak contact pressure of the medial compartment increased linearly with the increase of the varus angle. For each 1° increase in varus angle, the corresponding peak contact pressure increased by 0.46 MPa, which is consistent with the existing biomechanical data. In previous cadaveric studies, Mina et al. [11]reported that the average contact pressure of the medial compartment increased linearly with the increase of the varus angle. Willinger et al. [9] reported that 3°varus alignment increased the medial compartment pressure by 37.4%. Berk et al. [27] found in their finite element analysis that the average contact pressure of the medial compartment increased linearly with the increase of the varus angle. The above results demonstrate that our tibial varus knee FE model was reliable to demonstrate biomechanical changes to the knee joint.

Tibial varus knee is a common joint malalignment in clinical practice. Progressive increases in varus alignment alter tibiofemoral contact biomechanics, resulting in elevated peak contact pressure within the medial compartment. The study of Palmer et al. [28] has confirmed that the Medial Proximal Tibial Angle was significantly associated with the progression of osteoarthritis. For every one degree decrease (more varus) in the Medial Proximal Tibial Angle, the odds of joint space narrowing progression in the medial compartment increased by 21% (*p* < 0.001). The results of this study indicated the importance of varus knee angle in joint injury. Although osteoarthritis usually involves a varus angle greater than 10°, our model only covers mild varus knee (< 9°). However, the tibial varus knee model we established reveals that the varus angle affects the stress distribution on the meniscus tear surface from a mechanical mechanism, which may influence the meniscus healing potential and combine with the abnormal force line of the lower limbs to cause early osteoarthritis and aggravate it. Our study provided a new insight into the prevention of early osteoarthritis caused by meniscus tears in mild varus knee.

We conducted a numerical study on the finite element model of radial tears under the tibial varus knee. The study demonstrated a considerable increase in medial compartment peak contact stress following radial meniscal tears, which is consistent with the results of Zhang et al. [4] and Bedi et al. [29]. However, unlike previous studies, we demonstrated that varus angle had a limited role in exacerbating the stress difference between the tear apex and the intact meniscus. In radial tears, the hoop collagen fibers are disrupted even under normal alignment, and the load-bearing capacity is reduced regardless of the degree of varus alignment. This resulted in stress concentration at the tear apex, further propagating meniscus tearing. Stress concentration at the tear apex persists during varus knee, which may be the cause of the aggravation of the tear. The apex stress of unstable radial tears was higher than that of stable radial tears, which may be why the healing potential of longer meniscal tears is relatively low in clinical practice [30, 31].

Finite element analysis of longitudinal tears revealed that both stable and unstable tears at various locations under neutral alignment position significantly increased peak contact pressures in the medial compartment, which was confirmed by the findings of Chen et al. [32] and Muriuki et al. [33].Our study also found that longitudinal tears occurring in the red zone, red-white zone, and white zone of the meniscus have stress differences on both the inner and outer surfaces. Stable longitudinal tears of the meniscus have favourable stress differences. Kedgley et al. [7] found that the favourable stress difference generated by the meniscal tear surface compresses the tear surface together, and the favourable stress difference can promote healing potential. Their research suggested that biomechanics may play a key role in the meniscus healing process. Kijowski et al. [34] found that longitudinal tears less than 2 mm occurring at the junction of the meniscus and the joint capsule can heal spontaneously. Our findings suggest that enhanced healing capacity in this region may be attributed not only to its abundant vascular supply but also to the presence of favourable stress distributions. Concurrently, our study identified that unstable longitudinal tears were characterized by unfavourable stress differences, which may impede the healing process. Tschopp et al. [35] conducted a prospective clinical cohort study and found that knee malalignment (varus ≥ 5°) was an independent identified as a potential nonhealing risk factor of medial meniscal tears in the red and red-white zones, and varus malalignment increased the risk of nonhealing. Other clinical studies [36–38] have also found that tear length has a negative impact on meniscal healing, which is consistent with our results. However, the reasons were not further analyzed. Our analysis results show that it may be related to unfavourable stress differences.

This study uniquely demonstrates how increasing tibial varus angle transforms a favorable stress environment into an unfavorable one, particularly in the peripheral zones, which has not been quantitatively addressed before. For stable longitudinal tears, the stress difference on the tear surface at the red, red-white, and white zones varied with the varus knee angle change. Stable longitudinal tears at the red zone always exhibited a favourable stress difference at mild varus knee (0–9°), indicating that this type of patient with tibial varus knee might be most suitable for non-surgical treatment. Stable longitudinal tears at the red-white zone showed a reduced stress difference at 0–6° varus knee. Still, they maintained a favourable stress difference, indicating that this type of patient with tibial varus knee was also suitable for non-surgical treatment. Although there was a favorable stress difference in the stable longitudinal tear at the white zone, this area has no blood vessels and may not heal solely by biomechanical factors. With increasing varus knee angle, an unfavorable stress difference emerged on the surface of stable longitudinal tears at the red-white and white zones. This suggests to the clinician that even small and stable longitudinal tears may influence altered stress distribution due to varus knee angle and may influence the healing ability of the meniscus. Novaretti et al. [12]demonstrated that small longitudinal tears in the lateral meniscus significantly expand under valgus torque, influencing meniscal repair and subsequent healing. Their findings suggest that the varus alignment plays an important role in the healing potential of meniscal longitudinal tears. The analysis of unstable longitudinal tears indicated that unfavourable stress differences were observed across all varus knee angles. An unfavorable stress difference appears in the white zone, showing a varus angle-dependent increase. This suggests that tears in the white zone are more susceptible to the influence of tibial varus knee angle, potentially necessitating surgical intervention for unstable longitudinal tears in this region. In the treatment of meniscal injuries, the presence of varus alignment raises concerns about healing, regardless of whether conservative treatment or meniscal repair is selected. Therefore, it is important to not only that conservative treatment may be appropriate, but also that healing can be expected even with surgical repair.

Unlike other researchers, the ligaments and cartilage constructed in this study are hyperelastic isotropic materials, and the meniscus is a transversely isotropic hyperelastic material. By doing so, we can more accurately describe the correct mechanical properties of the knee joint. It is important to acknowledge the limitations of this study. First, the validity verification of the finite element model in this study only refers to the research data of others, lacking biomechanical cadaver data, which may affect the accuracy of the results. In view of the limitations of the research design, future work is needed for quantitative comparison (e.g., RMS error) with in-vitro pressure-sensor data from cadaveric experiments, meniscal strain gauges, or MRI-based cartilage contact maps and clinical data for validation. **S**econd, the potential influence of meniscal extrusion was not considered. Varus alignment may lead to meniscal protrusion, influencing the meniscal biomechanical environment. In the future, we suggest that meniscal extrusion be used as an influencing factor to study the stress distribution of the meniscus.

Third, this study only considered mild tibial varus knees and did not consider the types with greater angles of varus and other sources. In clinical practice, sources of varus knee also include femoral varus and mixed varus. Given the limited computing resources and the complexity of establishing varus knee models. Therefore, additional research is necessary to establish models from other sources and consider greater angles of the varus knee, providing new insights into the healing potential of meniscal tears.

In addition, this model does not include muscle forces. Muscle structure contributes to knee stability and may also influence the healing potential of meniscal tears. Related studies have found that incorporating muscle force into the knee joint model and applying accurate muscle force is challenging [39]. Many scholars have established knee joint models without including muscle forces but have obtained many acceptable outcomes [7]. In the future, we need to establish a musculoskeletal model to further study the impact of muscle force on the healing potential of meniscus. This study applied no fluid-pressurisation or time-dependent cartilage behaviour, and some researchers define cartilage materials as transversely isotropic materials, and more models need to be established in the future to compare the advantages and disadvantages. Finally, this study used a single-subject geometry to develop a finite element model. The evolution of the disease is affected by many factors, such as the individual variability or pathological conditions. Whether this conclusion is applicable to explain the intervention efficacy of different meniscus tear patients still needs to be further observed and confirmed. Future work should develop more knee joint models using a number of volunteers.

## Conclusions

To our knowledge, this study is among the first to investigate the influence of varus alignment on stress distribution in meniscal tears using finite element analysis. This study investigates two types of radial tears and two types of longitudinal tear prototypes in three zones simultaneously. This study provides novel insights into how varus alignment influences stress distribution in meniscal tears, suggesting its potential role in guiding treatment decisions. Future studies incorporating clinical data are warranted to validate these findings.

## Supplementary Information

Supplementary Material 1

## Data Availability

The datasets generated and/or analysed during the current study are not publicly available due privacy of the patient but are available from the corresponding author on reasonable request.

## Abbreviations

BMI: Body Mass Index
HKA: Hip-Knee-Ankle angle
mMPTA: Mechanical medial proximal tibial angle
mLDTA: Mechanical lateral distal tibial angle
mLDFA: Mechanical lateral distal femoral angle
JLCA: Joint line convergence angle
MRI: Magnetic resonance imaging
CT: Computed tomography
ACL: Anterior cruciate ligament
PCL: Posterior cruciate ligament
MCL: Medial collateral ligament
LCL: Lateral collateral ligament
3D: Three-dimensional
E: Elastic modulus
ν: Poisson's ratio
HGO: Holzapfel-Gasser-Ogden
