# Supplementary material for: Stability characteristics of medial meniscus tear in mild varus knee: a finite element analysis
Source: BMC Musculoskelet Disord. 2025 Oct 9;26:944. doi: 10.1186/s12891-025-09046-4 (PMC12512496; doi:10.1186/s12891-025-09046-4)
Supplement: Supplementary file 1 — Supplementary Material 1 [file 12891_2025_9046_MOESM1_ESM.docx]

Supplementary Material

**Articular Cartilage Material**

The strain energy function of Young's modulus was as described below:

$$\Phi=C_{10}\left(\bar{I}_{1}-3\right)+C_{20}\left(\bar{I}_{1}-3\right)^{2}+C_{30}\left(\bar{I}_{1}-3\right)^{3}+\frac{1}{D_{1}}(J-1)^{2}+\frac{1}{D_{2}}(J-1)^{4}+\frac{1}{D_{3}}(J-1)^{6}\text{ }\left(1\right)$$

Where: $\overline{I_{1}}$- the first invariant of the modified left Cauchy–Green tensor; J- the elastic volume ratio;$C_{10}$,$C_{20}$,$C_{30}$,$D_{1}$,$D_{2}$,$D_{3}$- independent Yeoh material constants. The values of the parameters $C_{10}$,$C_{20}$for normal and degenerated knee cartilage were taken from an experimental study [1]，see Table 1. However, the constant $C_{30}$was assumed to be zero due to a lack of experimental data [2].The values of the parameters$D_{1}$were calculated from the values of$C_{10}$Poisson's ratio of v =0.45 (Table 1).

**Table 1** Material parameters of normal and osteoarthritic articular cartilage [1]

| Cartilage | Tibial | Femoral |
| --- | --- | --- |
| $C_{10}$(MPa) | 2.0 | 1.4 |
| $C_{20}$ (MPa) | 4.5 | 3.6 |
| $D_{1}$ (MPa^-1^) | 0.0517 | 0.0739 |

**Meniscus Material**

Meniscus material model with the function constructed in the form [3]:

$U=C_{10}\left(\bar{I}_{1}-3\right)+\frac{1}{D_{1}}\left(\frac{\left(J_{el}\right)^{2}-1}{2}-\mathrm{In}J_{el}\right)+\frac{k_{1}}{2k_{2}}\left(\exp\left[k_{2}\left\langle\bar{E}_{a}\right\rangle^{2}\right]-1\right)$ (2)

With

$\bar{E}_{\alpha}=\kappa\left(\bar{I}_{1}-3\right)+(1-3\kappa)\left(\bar{I}_{4(\alpha\alpha)}-1\right)$ (3)

Where:$\bar{I}_{4(\alpha\alpha)}$- a pseudoinvariant of the symmetrically modified Cauchy-Green strain tensor, which simulates hard elastic collagen fibers. Among the parameters, $C_{10}$、$D_{1}$、$k_{1}$、$k_{2}$and$\kappa$（Table 2）are used by Abaqus software to simulate real hyperelastic material properties in the calculation.

**Table 2** Material parameters used for modeling the medial and lateral meniscus[3, 4]

| Component | $C_{10}(MPa)$ | $D_{1}(MPa^{-1})$ | $k_{1}$[-] | $k_{2}$[-] | $\kappa$[-] |
| --- | --- | --- | --- | --- | --- |
| Medial menisicus | 1 | 5e-3 | 5.0 | 0.9 | 0 |
| Lateal menisicus | 1 | 5e-3 | 8.5 | 1.6 | 0 |

Note: $C_{10}$and$D_{1}$:neo-Hookean constants; $k_{1}$和$k_{2}$: HGO coefficients; $\kappa$: Fiber dispersion and orientation level.

**Ligaments material**

Its strain energy function is[5]：

$\Phi=C_{1}\left(\bar{I}_{1}-3\right)+\frac{1}{D_{1}}(J-1)^{2}+F_{2}(\lambda), J=\det\mathbf{F}, \bar{I}_{1}=\mathrm{tr}\overline{\mathbf{B}}$ (4)

Contains the Neo-Hookean formula, Two of the constants $C_{1}$, and$D_{1}$（Table 3） defines the matrix substance and transverse part$F_{2}\left(\lambda\right)$ according to the stiffness of collagen fibers, and the function$F_{2}\left(\lambda\right)$ satisfies the conditions:

$\lambda\frac{\partial F_{2}(\lambda)}{\partial\lambda}=\left\{\begin{matrix} 0, & \lambda\leq1, \\ C_{3}\left(\exp\left(C_{4}(\lambda-1)\right)-1\right), & 1<\lambda<\lambda^{*}, \\ C_{5}\lambda+C_{6}, & \lambda\geq\lambda^{*}, \end{matrix}\right.$ (5)

Where$\lambda$ is the fiber stretch,$\lambda^{*}$is the limit value of stretch corresponding to straightened fiber,$C_{1}$is related to the shear modulus $u(C_{1}=2/u)$,$J$ is the determinant of the deformation tensor $F$, $\overline{I_{1}}$is the first invariant of the deviatoric left Cauchy-Green tensor $\overline{I_{1}}=tr\overline{FF}^{T}$with a deformation gradient of $\overline{F}\left(\overline{F}=J_{F}^{-0.33}F\right)$.

The stress in the fiber depends on the fiber stretch $\lambda$， which is determined by the deformed fiber orientation$a_{d}$, the deformation gradient$F$, and the initial fiber orientation$a_{0}\left(\lambda a_{d}=Fa_{0}\right)$. If the fiber is under compression$\lambda$≤ 1, it will not be subjected to any compressive stress. As the fiber is stretched between 1 and a predefined value ($\lambda$*), the stiffness of the fiber increases exponentially, beyond which the fiber straightens and the stiffness increases linearly.

Where:$C_{3}$- the exponential stress;$C_{4}$- the rate of collagen uncramping;$C_{5}$- the elastic modulus of the straightened collagen fibers;$C_{6}$- calculated from the continuity condition of $\lambda$, the constant $C_{6}$ is introduced to ensure that the stresses are continuous at$\lambda*\left[C_{6}=\left(e^{C_{4}\left(\lambda*-1\right)}-1\right)\bullet C_{3}-\left(C_{5}\lambda^{*}\right)\right]$. [6]

**Table 3** Material constants of the ligaments[5]

|  | $C_{1}\left(MPa\right)$ | $D_{1}\left(MPa^{-1}\right)$ | $C_{3}\left(MPa\right)$ | $C_{4}$[-] | $C_{5}\left(MPa\right)$ | $\lambda^{*}$[-] |
| --- | --- | --- | --- | --- | --- | --- |
| ACL | 1.95 | 0.01366 | 0.0139 | 116.22 | 535.039 | 1.046 |
| LCL | 1.44 | 0.00252 | 0.57 | 48.0 | 467.1 | 1.062 |
| MCL | 1.44 | 0.00252 | 0.57 | 48.0 | 467.1 | 1.062 |
| PCL | 3.25 | 0.0082 | 0.1196 | 87.178 | 431.063 | 1.035 |

Note: Constants $C_{1}$and $D_{1}$-Neo-Hookean constants,$C_{3}$- the exponential stress,$C_{4}$- the rate of collagen uncramping $C_{5}$- the elastic modulus of the straightened collagen fibers,$\lambda^{*}$- predetermined fiber stretch value beyond which collagen fibers straighten.

**References**

1. Robinson DL, Kersh ME, Walsh NC, Ackland DC, de Steiger RN, Pandy MG: Mechanical properties of normal and osteoarthritic human articular cartilage. J Mech Behav Biomed Mater. 2016, 61:96-109.<https://dx.doi.org/10.1016/j.jmbbm.2016.01.015>

2. Daszkiewicz K, Luczkiewicz P: Biomechanics of the medial meniscus in the osteoarthritic knee joint. PeerJ. 2021, 9:e12509.<https://dx.doi.org/10.7717/peerj.12509>

3. Xu Z, Li Y, Rao J, Jin Y, Huang Y, Xu X, Liu Y, Tian S: Biomechanical assessment of disease outcome in surgical interventions for medial meniscal posterior root tears: a finite element analysis. Bmc Musculoskelet Dis. 2022, 23(1):1093.<https://dx.doi.org/10.1186/s12891-022-06069-z>

4. Danso EK, Makela JT, Tanska P, Mononen ME, Honkanen JT, Jurvelin JS, Toyras J, Julkunen P, Korhonen RK: Characterization of site-specific biomechanical properties of human meniscus-Importance of collagen and fluid on mechanical nonlinearities. J Biomech. 2015, 48(8):1499-1507.<https://dx.doi.org/10.1016/j.jbiomech.2015.01.048>

5. Luczkiewicz P, Daszkiewicz K, Chroscielewski J, Witkowski W, Winklewski PJ: The Influence of Articular Cartilage Thickness Reduction on Meniscus Biomechanics. PloS one. 2016, 11(12):e0167733.<https://dx.doi.org/10.1371/journal.pone.0167733>

6. Li L, Yang L, Zhang K, Zhu L, Wang X, Jiang Q: Three-dimensional finite-element analysis of aggravating medial meniscus tears on knee osteoarthritis. J Orthop Translat. 2020, 20:47-55.<https://dx.doi.org/10.1016/j.jot.2019.06.007>
